# Bringing stakeholders together across ageing and disability: GOWD conference series

**DOI:** 10.5334/ijic.1083

**Published:** 2012-11-16

**Authors:** Andria Spindel, Margaret Campbell, Jennifer Mendez

**Affiliations:** March of Dimes Canada/Ontario, Canada; National Institute on Disability and Rehabilitation Research, Washington, DC, USA; School of Medicine, Wayne State University, Detroit, Michigan, USA

Jeannette Shannon was 69 in 2002, and chair of March of Dimes Canada (MODC). Disabled from Polio, Jeannette had been experiencing significant health problems and concluded that people with disabilities who often experienced early ageing-related conditions were sometimes ignored by the fields of both rehabilitation and geriatrics as they aged, and had complex care issues related to existing disabilities and new health concerns. Known as Mrs. March of Dimes, Ms. Shannon passed away before she could see the result of her vision: to deliver a conference on “growing older with a disability,” bringing consumers of health and social services together with practitioners, policy-makers, researchers and businesses. In 2007 and 2011 Ms. Shannon’s vision came to fruition through the establishment of the Growing Older with Disability (GOWD) conferences and the larger umbrella framework of which they are a central component, namely the Festival of International Conferences on Caregiving, Disability, Aging and Technology (FICCDAT).

## Creation of GOWD and FICCDAT

In 2004, MODC Board approved Ms. Shannon’s plan to create an ageing and disability conference. Within a year the agency had funding from the Ontario provincial Seniors’ Secretariat to implement this plan and had partnered with the 2nd International Conference on Technology and Aging (ICAT), which was developed by the Toronto Rehabilitation Institute to expand the focus to include technology. Consultations with many community support agencies and researchers confirmed the view that the health and community living needs of individuals with long-term disabilities were increasing and becoming more complex, thus bringing new and existing recipients into the system with increased service demands, requiring more service hours and interventions. Moreover, annual increases in provincial government funding of community support agencies were not keeping pace with the number and complexity of service needs of people ageing with long-term disability, thus raising the threat of unnecessary and increased hospitalizations.

Faced with the changing realities of increased longevity within the disabled population, a collaboration was formed under the leadership of MODC to develop the Growing Older with a Disability conference with the goal of identifying creative solutions, new models for service, partnerships, and new policy options with government that support persons ageing with disabilities. In addition, the chair of ICAT reached out to colleagues within the University of Toronto and found other departments eager to host academic meetings that would be cross disciplinary while considering consumer input and feedback. Thus the Festival of International Conferences on Caregiving, Disability, Aging and Technology (FICCDAT) was born, which served as an umbrella framework for multiple inter-related concurrent conferences on disability, ageing, technology, and caregiving. In 2011 the following conferences were offered with shared social and educational programs: Growing Older with a Disability; Advancing Rehabilitation Technologies for an Aging Society; Advances in Neurorehabilitation; Caregiving in the 21st Century; 34th Canadian Medical and Biological Engineering Conference and International Conference on Best Practices in Universal Design. This unique infrastructure has provided support for international dialogue across these issues.

In 2007 the first FICCDAT, offering five concurrent conferences, was successfully launched in Toronto, Canada, and attended by over 1300 people from 39 countries. The second FICCDAT was held in 2011, also in Toronto, offered six concurrent conferences and attracted nearly as many people from North America, Europe, Australia, Africa, Asia and the Middle East. Both festivals were planned and coordinated by an international Steering Committee of ageing and disability representatives from government agencies, businesses, NGOs, and academic communities, and included consumers and caregivers. The purpose of the Steering Committee was to generate commitment and ‘ownership’ of the Festival, identify qualified presenters, promote international participation, share liability, reduce dependence on external funds, facilitate the production of Festival outputs that disseminate conference results, and build international awareness around critical issues related to ageing with a disability [1].

## Outcomes of GOWD and FICCDAT

Together, the FICCDAT 2007 and 2011 demonstrated the critical need for a platform to support cross-disciplinary, international exchange of knowledge on issues related to ageing with disability. The process of bringing stakeholders together across ageing and disability lines has been referred to as ‘bridging’ by a cadre of thought leaders in Europe, the US, and Canada [2, 3]. Formally defined in the Toronto Declaration [4] bridging refers to bringing networks of stakeholders and streams of knowledge, practice and policy together to close gaps in understanding, data and services to better meet the health and support needs of the rapidly expanding world-wide population of people who are both ageing with and ageing into long-term disabilities in mid- and later life.

The 2007 GOWD meeting identified the need to continue and expand the conversation and to create momentum for an international initiative aimed at bridging knowledge, practice and policy across traditional ageing and disability lines. In 2011, plans were made to produce a unified statement based on GOWD presentation findings in order to formally support development of research, policy, and practice related to ageing with disability. This statement is the Toronto Declaration (TD) on Bridging Knowledge, Practice and Policy in Aging and Disability, developed by an expert panel of international researchers, policy-makers and service providers [4] who presented at GOWD. It addresses the need for collaboration among stakeholders and sets out recommendations that will foster improvements in policies and care, emphasizing similarities in experiences and needed supports, services and policies, rather than focusing on differences. The Declaration has been translated into French, German, Hebrew, Japanese, Mandarin, Russian, Spanish, and Urdu. To publicize the TD and seek support, a number of international presentations were made at both community service and scientific organizations. Other achievements from the conference are two reports based on the GOWD conference funded by the Canadian Office of Disability Issues and a third report funded by MODC is based on the Caregiving conference. These reports identify policy, practice, and research recommendations based on conference presentations and a review of existing scholarly literature.

The relevance of the Toronto Declaration was highlighted in recent months as new developments within the US evolve on bridging ageing and disability knowledge and practice. These include a first ever 2012 interagency conference on ageing with disability, jointly sponsored by three leading US research and policy agencies on ageing and disability [5] and the establishment in 2012 of a new Institute of Medicine Forum on Aging, Disability and Independence, dedicated to providing a neutral venue for broad ranging discussions, coordination and integration of ageing and disability stakeholders [6]. With the ultimate goal to serve all individuals with respect to long-term supports and services preferences regardless of age or disability, the Forum will highlight capacities in which ageing and disability network coordination is strong; examine the historic challenges faced by aligning the ageing and disability networks; explore new approaches for resolving problem areas; elevate the visibility and perspectives of the many relevant stakeholders; and set the stage for future policy actions [6]. These initiatives are just two promising examples of government attention to bridging, but they each draw attention to bridging and thus have potential to contribute to national and international agendas on bridging.

Having developed a strong model for bringing ageing and disability stakeholders together, the GOWD conferences and the larger FICCDAT umbrella will continue to be important for working across network lines to help close the gaps in age- and disability-based services and to curtail policies that negatively affect the lives of the expanding population of people ageing with long-term developmental, physical, cognitive and sensory disabilities, as well as those ageing into disability in mid- to later life. Additionally, GOWD and FICCDAT remain important contributors to the international exchange of ideas, data and best practices between ageing and disability.

## The role of GOWD and FICCDAT going forward

While a few government agencies in Europe and North America, such as the Executive Agency of Health and Consumers (EAHC) within the European Commission and the National Institute on Disability and Rehabilitation Research (NIDRR) within the U.S. Department of Education (NIDRR), have a history of funding research on ageing with disability, prior to GOWD and FICCDAT there was no ageing or disability organizational ‘home’ and little in the way of sustained momentum to support bridging knowledge across ageing and disability network lines. Over the same period of time, there also have been a number of high-profile conferences in Europe and North America on ageing with disability that produced considerable excitement and numerous scientific publications. Examples include the series of International Conference on Aging, Disability and Independence [7–10] and the Barcelona Conference on Bridging and Knowledge Transfer of 2009 [11]. However, to date, none of these efforts have gained much traction within the traditional disciplines of disability and ageing, namely rehabilitation and gerontology, or resulted in much needed increased funding for ageing with disability research [2, 3, 12]. Thus, up until very recently, the history of bridging efforts has remained on the sidelines of mainstream ageing and disability research, policy and practice without a governmental or organizational home, sustained commitment, and international champions.

March of Dimes of Canada and the Toronto Rehabilitation Institute envisioned the FICCDAT meetings as a response to this lack of institutional organization and sponsorship in facilitating the work of bridging ageing and disability. MODC continues to support the GOWD Expert Panel collaboration, disseminating information to past GOWD participants through its website, and is planning for a third FICCDAT in 2015. Despite the considerable accomplishments of GOWD and FICCDAT, there is much more that needs to be done to turn the vision of bridging into a sustainable focus of international research, practice and policy development that delivers real benefits for the millions of people around the world ageing with disability.

Potential future activities that could contribute to this goal include expanding existing cross-national partnerships, and leveraging the Toronto Declaration to create a formal international network on bridging disabilities and ageing. Such a network could bring international thought leaders and advocates together to build the organizational and scientific infrastructure necessary to support the development of a research agenda and action plan to promote the generation, exchange and use of knowledge and best practices to improve the lives of the expanding population of people ageing with and into disability around the world. FICCDAT, GOWD and MODC will continue to contribute to this work.
